# NY-ESO-1 antigen-reactive T cell receptors exhibit diverse therapeutic capability

**DOI:** 10.1002/ijc.27792

**Published:** 2012-08-21

**Authors:** Daniel Sommermeyer, Heinke Conrad, Holger Krönig, Haike Gelfort, Helga Bernhard, Wolfgang Uckert

**Affiliations:** 1 Max-Delbrück-Center for Molecular MedicineBerlin, Germany; 2 Division of Immunogenetics, German Cancer Research Center (DKFZ)Heidelberg, Germany; 3 Department of Hematology/Oncology, Klinikum rechts der Isar, Technische Universität MünchenMunich, Germany; 4 Department of Hematology/Oncology, Klinikum Darmstadt GmbHDarmstadt, Germany; 5 Institute of Biology, Humboldt University BerlinBerlin, Germany

**Keywords:** tumor immunity, human, cytotoxic T cells, T cell receptor, NY-ESO-1

## Abstract

The cancer-testis antigen NY-ESO-1 has been used as a target for different immunotherapies like vaccinations and adoptive transfer of antigen-specific cytotoxic T cells, as it is expressed in various tumor types and has limited expression in normal cells. The *in vitro* generation of T cells with defined antigen specificity by T cell receptor (TCR) gene transfer is an established method to create cells for immunotherapy. However, an extensive characterization of TCR which are candidates for treatment of patients is crucial for successful therapies. The TCR has to be efficiently expressed, their affinity to the desired antigen should be high enough to recognize low amounts of endogenously processed peptides on tumor cells, and the TCR should not be cross-reactive to other antigens. We characterized three NY-ESO-1 antigen-reactive cytotoxic T lymphocyte clones which were generated by different approaches of T cell priming (autologous, allogeneic), and transferred their TCR into donor T cells for more extensive evaluations. Although one TCR most efficiently bound MHC-multimers loaded with NY-ESO-1 peptide, T cells expressing this transgenic TCR were not able to recognize endogenously processed antigen. A second TCR recognized HLA-A2 independent of the bound peptide beside its much stronger recognition of NY-ESO-1 bound to HLA-A2. A third TCR displayed an intermediate but peptide-specific performance in all functional assays and, therefore, is the most promising candidate TCR for further clinical development. Our data indicate that multiple parameters of TCR gene-modified T cells have to be evaluated to identify an optimal TCR candidate for adoptive therapy.

**What's new?**

The cancer-testis antigen NY-ESO-1 is expressed on many solid and hematological cancers but rarely in normal tissues and if so, in immune-privileged germ cells. To test its therapeutic potential as a target for immunotherapies, the authors characterized three different NY-ESO-1-specific T cell receptors, which were isolated from T cell clones generated by autologous and allogeneic approaches. They find considerable differences in TCR behavior towards MHC-associated NY-ESO-1 peptides, underscoring that multiple functional parameters of TCR-transgenic T cells have to be thoroughly characterized prior to clinical application of NY-ESO-1 directed immunotherapies.

The cancer-testis antigen NY-ESO-1 is an attractive target for specific immunotherapies, as it is expressed in many different solid tumors and hematological malignancies.^1^ In addition, the expression in normal tissue seems to be mainly restricted to immune privileged germ cells. NY-ESO-1 was originally identified by serological analysis of recombinant cDNA expression libraries using tumor mRNA and autologous serum from a patient with esophageal squamous cell carcinoma,^2^ indicating that a humoral immune response can spontaneously arise in tumor patients. Moreover, NY-ESO-1 epitopes for CD4^+^ and CD8^+^ T cells were identified and these T cells were found in many patients with NY-ESO-1^+^ tumors.^3^, ^4^ Due to the proven immunogenicity of NY-ESO-1 several vaccination studies were performed. Although an increase in NY-ESO-1-specific T cells was observed in vaccinated patients, the clinical benefit was limited.^5^ One reason for the poor reactivity of T cells after vaccination might be the absence of high avidity T cells as these cells are probably deleted in the thymus due to NY-ESO-1 expression in medullary thymic epithelial cells.^6^ A possibility to circumvent this problem is the adoptive transfer of high avidity NY-ESO-1-specific T cells into patients. To reproducibly generate these T cells for every patient, autologous T cells can be modified with genes encoding for high affinity T cell receptors (TCRs). In a recent clinical study using this approach, nine of 17 patients showed clinical responses without serious side effects,^7^ confirming that NY-ESO-1 is a suitable antigen for adoptive T cell therapies. For a successful therapy with TCR gene-modified T cells it is crucial to use TCR with sufficiently high affinities toward their antigen. TCR genes can be isolated from tumor infiltrating lymphocytes, which have the advantage that they have been primed *in vivo*. However, often low avidity T cells have been isolated, because tumor-associated antigens, such as NY-ESO-1 are also expressed by normal tissues including the thymus. A second source for TCR is T cells which were generated by “reverse immunology.” For this approach, an antigen is chosen and then peripheral blood lymphocytes (PBL) from a—in most cases healthy—donor are stimulated with this antigen to induce proliferation of T cells with the desired specificity. These cells can then be enriched and cloned by limiting dilution.^8^ To induce high avidity T cells against self antigens, PBL from allogeneic donors can be used.^9^ In this case an HLA mismatch between the T cells and the antigen presenting cells allows the isolation of unselected T cells. However, this approach bears the risk that the antigen recognition of the isolated TCR is dominated by the binding to the foreign major histocompatibility complex (MHC) molecule.^10^ So far, only NY-ESO-1-specific TCR-transgenic (TCR-tg) T cells with TCR originating from autologous approaches have been described.^11^, ^12^ In this study, we have characterized three different NY-ESO-1-specific TCR, which were isolated from T cell clones generated by autologous and allogeneic approaches in order to evaluate them for their potential therapeutical usage.

## Material and Methods

### Cells

Cell lines 293T (ATCC: CRL-11268, American Type Culture Collection, Manassas, VA), SK-Mel-29,^13^ SK-Mel-37,^13^ Mel-285 (B. Ksander, Schepens Eye Institute, Boston, MA), Me-324^14^ and MUC-Mel-1^15^ were cultured in Dulbecco's modified Eagle's medium (GIBCO, Karlsruhe, Germany) supplemented with 10% fetal calf serum (Biochrom AG, Berlin, Germany) and 100 U/ml penicillin/streptomycin. The renal cell carcinoma cell lines KT-195-VC and KT-195-A2^16^ were cultured in RPMI 1640 medium (GIBCO) supplemented with 10% fetal calf serum, 1 mM sodium pyruvate (GIBCO), and 100 U/ml penicillin/streptomycin. NY-ESO-1-expressing SK-Mel-29 cells (SK-Mel-29-NY) were generated by transducing them with the retroviral vector MIG containing the NY-ESO-1 gene. In parallel, cells were transduced with an empty MIG vector (SK-Mel-29-VC). Both cell populations were enriched for transduced cells by fluorescence activated cell sorting (FACS) based on green fluorescence protein (GFP) expression.

PBL were isolated from blood of healthy donors with donors' informed consent by ficoll gradient centrifugation and seeded in a concentration of 1 × 10^6^ per well and ml in the presence of 100 U/ml interleukin-2 (kindly provided by Chiron, Marburg, Germany) in non-tissue culture 24-well plates, precoated with anti-CD3 (5 μg/ml) and anti-CD28 (1 μg/ml) monoclonal antibodies (mAb) (BD Pharmingen, Heidelberg, Germany) for stimulation. On day 13 after isolation, PBL were rested for two days by reducing the interleukin-2 concentration to 10 U/ml. Human PBL, Jurkat76/CD8 (J76/CD8) and T2 (ATCC: CRL-1992) cells were cultured in RPMI 1640 medium supplemented with 10% fetal calf serum (PAN Biotech, Aidenbach, Germany), 1 mM HEPES and 100 U/ml penicillin/streptomycin. All cell culture flasks and plates were purchased from BD Falcon (Franklin Lakes, NJ).

### Generation of NY-ESO-1-specific T lymphocytes

The study was done in accordance with the precepts established by the Helsinki Declaration and approved by the Ethics Committee of the Technical University of Munich, Germany. The generation of NY-ESO-1-specific cytotoxic T lymphocyte (CTL) clones ThP2 and CM26 was described previously.^17^ For generation of CTL clone HL-2, monocyte-derived dendritic cells (DC) of an HLA-A2^+^ donor were loaded with 5 μg/ml NY-ESO-1_157-165_-peptide (NY-ESO-1_157-165_) and used for stimulation of autologous CD8^+^ T cells. Following two restimulations, HLA-A2/NY-ESO-1_157-165_-multimer^+^ (NY-multimer^+^) CD8^+^ T cells were sorted using a Moflo cell sorter (Cytomation, Fort Collins, CO). Subsequently, sorted T cells were cloned by limiting dilution.^18^ Two weeks later, clones were screened in a chromium release assay and NY-ESO-1-reactive clones were expanded as described.^19^

### Construction of retroviral vectors

To identify the sequences of the TCR genes, a 5′-RACE-PCR (GeneRacer Kit, Invitrogen, Karlsruhe, Germany) amplifying the variable regions of the TCRα- and TCRβ-chains including CDR3 was performed with RNA isolated from the T cell clones (Rneasy Mini Kit, Qiagen, Hilden, Germany). RACE-PCR products were sequenced. The sequence of both TCR constant regions were exchanged by minimally murinized variants^20^ and mutated to create an additional disulfide bond.^21^, ^22^ TCRα- and TCRβ-chains were linked by a 2A peptide linker (TCRβ-P2A-TCRα)^23^ and the complete constructs were codon-optimized^24^ and synthesized (GENEART, Regensburg, Germany). The synthesized fragments were cloned into the retroviral vector plasmid pMP71 *via* NotI and EcoRI restriction sites.^25^

The NY-ESO-1 gene was amplified by PCR (5′-primer: TTTAGATCTGCCACCATGCAGGCCGAAGGCCG, 3′-primer: AAGAATTCATTAGCGCCTCTGCCCTGAGGG and cloned into the retroviral vector plasmid pMIG^26^*via* BglII and EcoRI restriction sites.

### Generation of retroviruses and transduction

Amphotropic MLV-10A1-pseudotyped retroviruses were produced as described.^20^ Forty-eight hours after transfection, viral supernatants were filtrated (0.45 μm pore size) and used directly for transduction. J76/CD8 cells (2 × 10^5^ per well) were incubated in 24-well non-tissue culture plates precoated with RetroNectin (3.5 μg/well) (Takara, Apen, Germany) with 1 ml retrovirus supernatant supplemented with protamine sulfate (final concentration 4 μg/ml) (Sigma-Aldrich, Munich, Germany). After addition of supernatant, plates were spinoculated with 800*g* for 1.5 hr at 32°C. PBL were transduced on day two and three after isolation as described.^23^ The adherent cell line SK-Mel-29 was seeded into 24-well-tissue culture plates one day before transduction (5 × 10^4^ cells per well). For transduction, 1 ml retrovirus supernatant supplemented with protamine sulfate (final concentration 4 μg/ml) was added.

### Flow cytometry

Cells were stained using fluorescein-isothiocyanate (FITC)-labeled mAb directed against human CD8 (BD Pharmingen), phycoerythrine (PE)-labeled mAb directed against human CD3 (BD Pharmingen) and allophycocyanin (APC)- or PE-labeled NY-multimers (Coulter, Krefeld, Germany and Dirk Busch, TU Munich, Germany, respectively). Fluorescence intensity was measured using a FACSCalibur flow cytometer (BD) and Cellquest Pro software (BD). Data were analyzed using FlowJo software (Tree Star, Ashland, OR).

### Cytokine release assay

TCR-tg PBL (1 × 10^5^ per well) were co-cultured with 5 × 10^4^ target cells in 200 μl medium. PBL cultured without target cells and PBL stimulated with phorbol 12-myristate 13-acetate (PMA)/ionomycin were used as negative and positive controls, respectively. Peptides were synthesized by Metabion (Martinsried, Germany) and loaded on T2 cells in a concentration of 10 μM. Supernatants from co-cultured cells were obtained after 24 hr and analyzed for content of human interferon-γ (IFN-γ) by ELISA (BD Biosciences, Heidelberg, Germany). IFN-γ concentrations are given as mean values of duplicates with mean deviation.

### Cytotoxicity assay

Cytolytic activity of CTL clones and TCR-tg PBL against different target cell lines was determined in 4 hr chromium release assays as described previously.^15^ To determine the functional avidity, T cells were incubated with 1 × 10^3^ for CTL clones or 2 × 10^3^ for TCR-tg PBL NY-ESO-1_157-165_-loaded (10^−12^ to 10^−6^ M) T2 cells. Specific and relative lysis was calculated from duplicates at each E/T or peptide concentration as described.^8^

## Results

### Auto- and allo-HLA-A2-restricted CTL clones recognize the same NY-ESO-1-derived epitope but differ in their multimer binding and functionality

**Figure 1:**
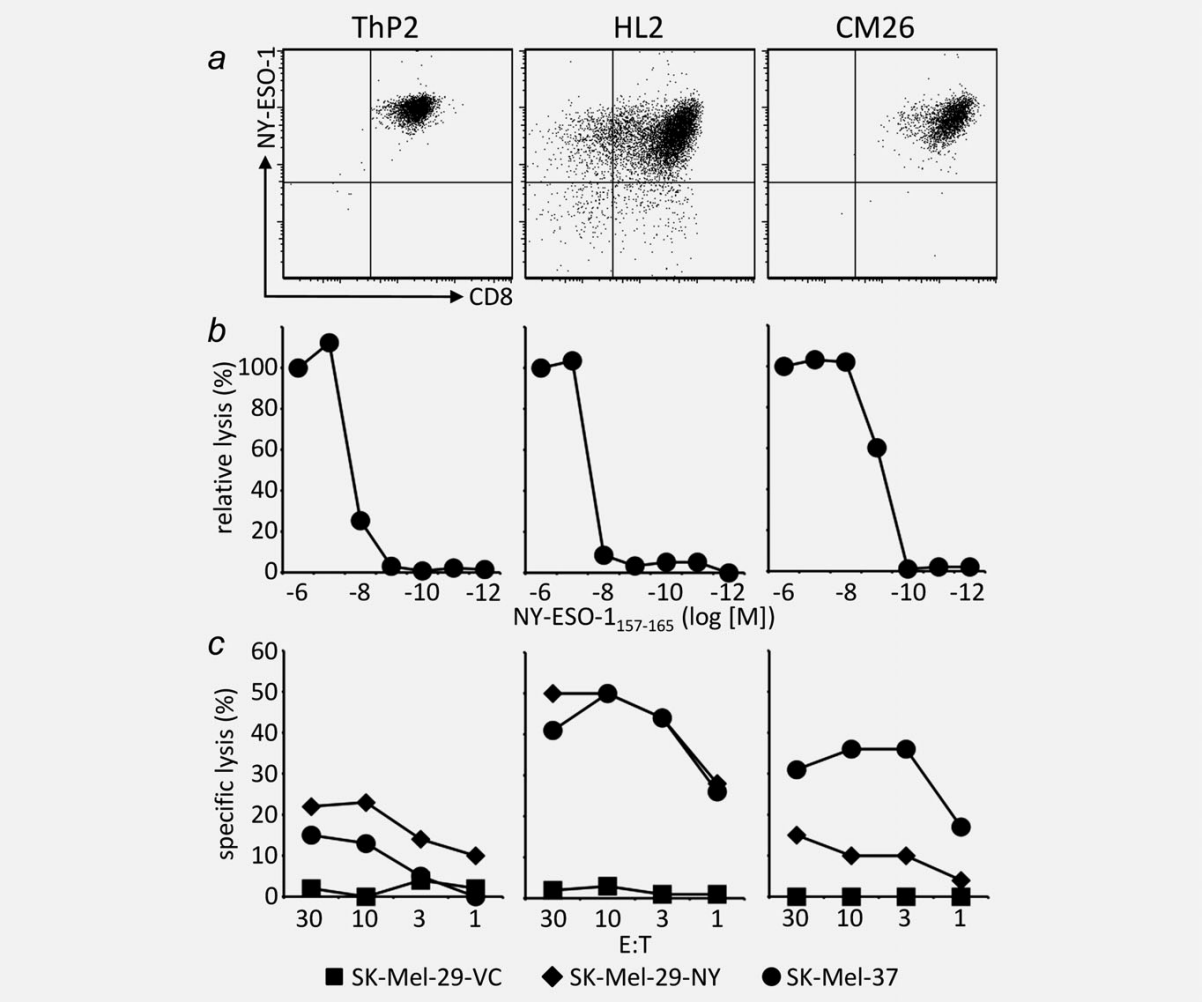
Auto- and allo-HLA-A2-restricted NY-ESO-1-reactive CTL clones differ in their functionality. T cells from HLA-A2^+^ and HLA-A2^−^ donors were stimulated with autologous and allogeneic HLA-A2^+^ NY-ESO-1_157-165_-loaded DC, respectively, and T cell clones were generated by limiting dilutions. (*a*) Isolated clones were stained with anti-CD8 mAb and NY-multimers and analyzed by flow cytometry. (*b*) Cytolytic activity against T2 cells loaded with different concentrations of NY-ESO-1_157-165_ was measured. The specific lysis at 1 μM was set as 100%. (*c*) Cytolytic activity against NY-ESO-1^−^ SK-Mel-29-VC and NY-ESO-1^+^ SK-Mel-29-NY and SK-Mel-37 melanoma cell lines was measured at different E:T.

We generated NY-ESO-1-specific CTL clones by two different strategies: DC from an HLA-A2^+^ donor were loaded with NY-ESO-1_157-165_ and used for stimulation of autologous and HLA-A2^−^ allogeneic T cells, respectively. The autologous approach was performed with cells from two different donors and peptide concentrations of 200 μg/ml and 5 μg/ml. CTL clones from all three different approaches were analyzed for NY-ESO-1_157-165_ recognition and specific lysis of NY-ESO-1^+^ tumor cell lines (data not shown). From each approach the clone with best function based on killing of tumor cell lines was chosen: CM26 for the allogeneic approach, ThP2 for the autologous approach with 200 μg/ml peptide and HL2 for the autologous approach with 5 μg/ml peptide. All three clones were CD8^+^ and bound NY-multimers (Fig. 1*a*). Moreover, NY-ESO-1_157-165_-loaded T2 cells were lysed by all three clones, with the highest peptide sensitivity for the allo-HLA-A2-restricted clone CM26 (Fig. 1*b*). T2 cells loaded with control peptides were not lysed (data not shown). Also endogenously processed antigen recognized as HLA-A2^+^/NY-ESO-1^+^ tumor cell lines (SK-Mel-37 and SK-Mel-29-NY) were lysed. In the experiment shown in Figure 1*c*, the CTL clone CM26 displayed a relatively poor tumor cells lysis, which is not representative for this CTL clone and might be due to poor T cell fitness in this particular experiment. There was no killing of HLA-A2^+^/NY-ESO-1^−^ tumor cell lines (SK-Mel-29-VC) (Fig. 1*c*) or HLA-A2^−^/NY-ESO-1^+^ tumor cell lines (data not shown).

### Functionality and MHC-multimer binding of NY-ESO-1 TCR do not correlate

**Figure 2:**
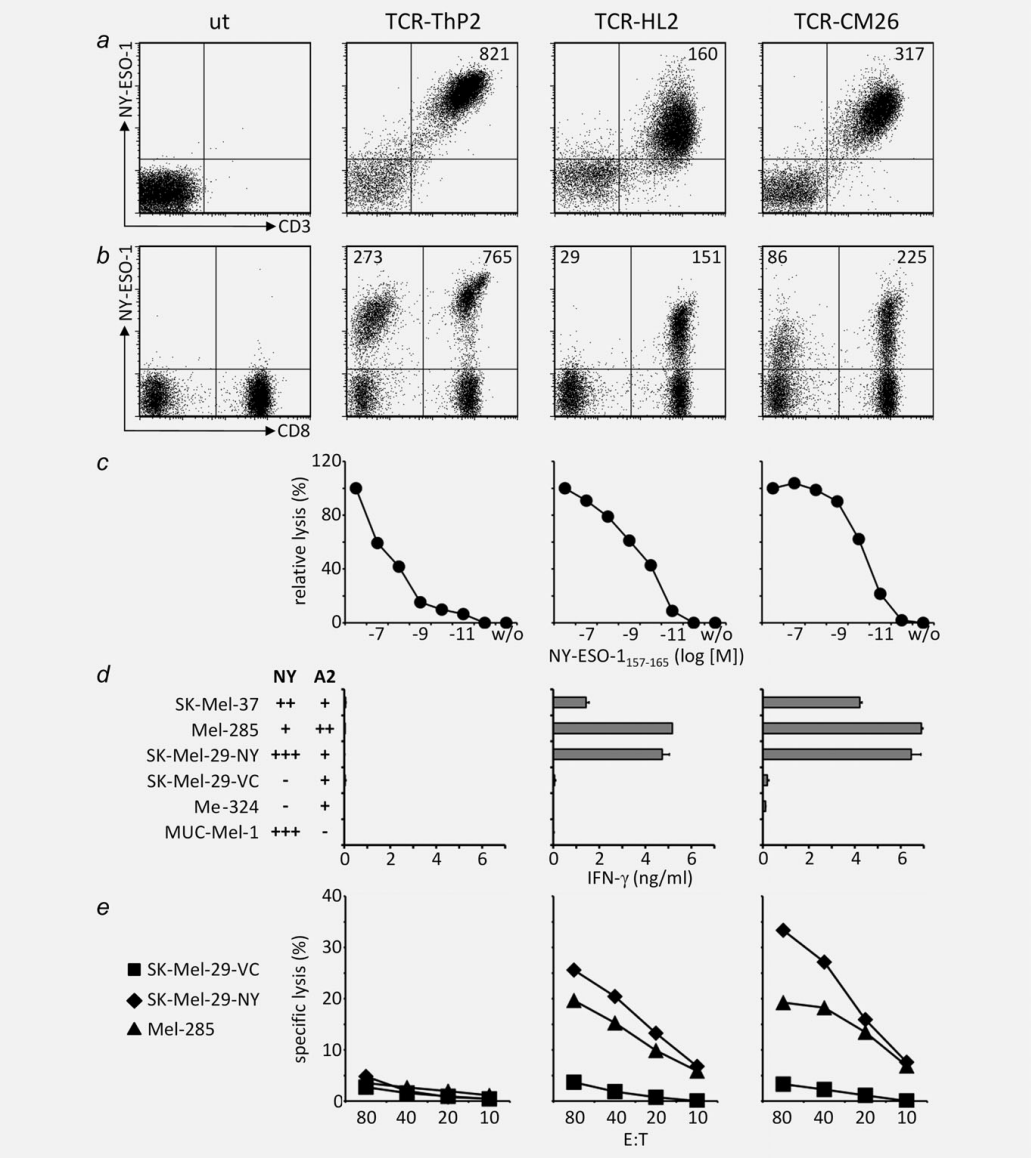
No correlation between the level of multimer binding and the functionality of TCR-modified T cells. (*a*) CD8α^+^ Jurkat 76 cells (J76/CD8) were transduced with optimized versions (minimally murinized, additional disulfide bond, codon-optimized) of NY-ESO-1_157-165_-reactive TCR-ThP2, TCR-HL2 and TCR-CM26. Four days after transduction, cells were stained with anti-CD3 mAb and NY-multimers and analyzed by flow cytometry. Numbers indicate the MFI of multimer staining for CD3^+^/multimer^+^ cells. Untransduced cells were used as a control (ut). (*b*) PBL from a healthy donor were transduced using the same viral supernatants as for J76/CD8 cells. Thirteen days after isolation, cells were stained with anti-CD8 mAb and NY-multimers and analyzed by flow cytometry. MFI of multimer staining for CD8^+^/multimer^+^ cells and CD8^−^/multimer^+^ cells are indicated. (*c*) Fifteen days after isolation, cytolytic activity against NY-ESO-1_157-165_-loaded T2 cells was measured at an E:T of 80:1. The specific lysis at 1 μM was set as 100%. (*d*) Transduced T cells were co-cultured with different melanoma cell lines and concentrations of released IFN-γ were analyzed by ELISA. Expression of NY-ESO-1 protein (NY) and HLA-A2 (A2) are indicated. (*e*) Cytolytic activity against melanoma cell lines was measured at different E:T.

To further characterize the TCR expressed by the three CTL clones ThP2, HL2 and CM26, we isolated the TCRα and TCRβ genes and cloned them into the retroviral vector MP71 as optimized variants linked by a viral peptide element (P2A). The retroviral vectors were used to transduce the TCR-deficient cell line J76/CD8 and primary human PBL. On both cell types, all three TCR were efficiently expressed (Figs. 2*a* and 2*b*). However, substantial differences in the capacity to bind NY-multimers were detected. On J76/CD8 cells the mean fluorescence intensity (MFI) of multimer staining was two times higher for TCR-CM26 (MFI: 317) and more than five times higher for TCR-ThP2 (MFI: 821) compared to TCR-HL2 (MFI: 160) which showed the lowest multimer binding (Fig. 2*a*). These differences were not due to an unequal TCR expression as the levels of CD3 were comparable for all TCR. None of the J76/CD8 cell lines bound HLA-A2 control multimers (Supporting Information Fig. 1). On PBL, the same hierarchy concerning multimer binding was observed. TCR-HL2-tg T cells showed the lowest multimer binding on CD8^+^ cells and there was no multimer binding by CD8^−^ cells (Fig. 2*b*). For TCR-CM26 and TCR-ThP2 multimer binding on CD8^+^ and CD8^−^ cells was observed with higher levels for TCR-ThP2 on both cell types. These results suggested that TCR-ThP2 had a higher affinity compared to TCR-HL2 and TCR-CM26 and should therefore result in T cells with an increased functionality. To test this, the cytotoxic capacity of TCR-tg T cells against T2 cells loaded with titrated amounts of NY-ESO-1_157-165_ was analyzed. However, in contrast to the data obtained for multimer binding, TCR-ThP2-tg cells showed the lowest functional avidity as only high peptide concentrations were recognized. TCR-HL2-tg and TCR-CM26-tg T cells revealed a more than 100-fold higher functional avidity compared to TCR-ThP2-tg cells (Fig. 2*c*). The discrepancy between efficient multimer binding and cell lysis implies that the prediction of TCR quality based only on multimer binding is not sufficient.

### Recognition of antigen-expressing tumor cells is restricted to NY-ESO-1 TCR with moderate levels of multimer binding

To analyze whether TCR-tg T cells were able to recognize cells presenting endogenously processed antigen, we co-cultured them with different melanoma cell lines and analyzed the concentrations of released IFN-γ. TCR-ThP2-tg T cells did not recognize any of the melanoma cell lines independent of HLA-A2 and NY-ESO-1 expression (Fig. 2*d*). To exclude that these cells were intrinsically not able to produce IFN-γ, TCR-ThP2-tg T cells were stimulated unspecifically (PMA/Ionomycin) and with NY-ESO-1_157-165_-loaded T2 cells, respectively. After both types of stimulation, IFN-γ was released (data not shown). T cells transduced with TCR-HL2 and TCR-CM26 released up to 5.2 and 6.9 ng/ml IFN-γ, respectively, when they were co-cultured with HLA-A2^+^/NY-ESO-1^+^ melanoma cell lines. Cell lines with high HLA-A2 levels (Mel-285) or cells overexpressing NY-ESO-1 (SK-Mel-29-NY) (Supporting Information Fig. 2) were recognized most efficiently. No or little amount of IFN-γ was released after co-cultivation with HLA-A2^−^ (MUC-Mel-1) or NY-ESO-1^−^ (SK-Mel-29-VC, Me-324) melanoma cell lines. When HLA-A2^+^/NY-ESO-1^−^ cell lines were exogenously loaded with NY-ESO-1_157-165_ all TCR-tg T cells recognized these cells (data not shown). To further characterize the TCR-tg T cells, we tested their cytotoxic activity against different melanoma cell lines. As before, TCR-ThP2-tg T cells were not able to recognize antigen-positive melanoma cells. Only TCR-HL2-tg and TCR-CM26-tg T cells lysed HLA-A2^+^/NY-ESO-1^+^ cell lines (SK-Mel-29-NY, Mel-285) (Fig. 2*e*).

Examining the recognition of melanoma cells lines by TCR-transduced T cells confirmed the results from the peptide titration assay. The TCR with best multimer binding but lowest functional avidity (TCR-ThP2) showed no recognition of endogenously processed antigen.

### A NY-ESO-1 TCR of allogeneic origin shows off-target reactivity

**Figure 3:**
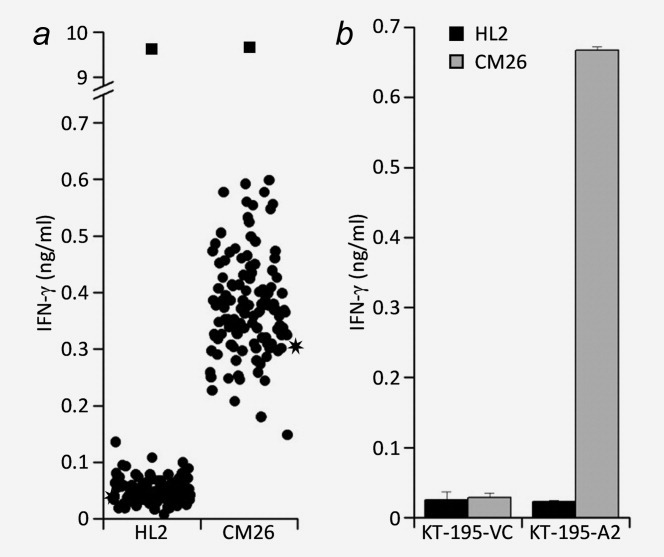
Peptide-independent HLA-A2 recognition by the allogeneicly generated TCR. (*a*) PBL from a healthy donor were transduced with optimized versions of NY-ESO-1_157-165_-reactive TCR-HL2 and TCR-CM26. Fifteen days after isolation, TCR-tg T cells were co-cultured with an array of T2 cells pulsed with 125 different HLA-A2-binding peptides, including NY-ESO-1_157-165_ (squares), or with unpulsed T2 cells (stars). Concentrations of released IFN-γ were determined by ELISA. (*b*) Transduced PBL were co-cultured with the HLA-A2^−^/NY-ESO-1^−^ renal cell carcinoma line KT-195 transduced with control vector (VC) or HLA-A2-containing vector (A2). Concentrations of released IFN-γ were analyzed by ELISA.

As TCR-ThP2-tg T cells did not recognize endogenously processed antigen, we continued only with TCR-HL2 and TCR-CM26. To further analyze the specificity of these two TCR, we used a peptide library consisting of 125 peptides known to bind to HLA-A2. The peptides were loaded on T2 cells which were then used for co-cultivation with TCR-tg T cells. For TCR-HL2-tg T cells the concentration of released IFN-γ was at the detection limit for all peptides, except for the NY-ESO-1_157-165_ positive control (Fig. 3*a*), indicating that the TCR specifically recognized only the desired peptide. In contrast, TCR-CM26-tg T cells showed increased recognition for all peptides with a mean IFN-γ release of 375 pg/ml. Although the amount of released IFN-γ was more than 25 times lower than after stimulation with the specific peptide, the TCR-CM26-tg cells seemed to react against the HLA-A2^+^ T2 cells independent of the bound peptide (Fig. 3*a*). To further specify the recognition of HLA-A2 by TCR-CM26-tg T cells, we co-cultured these cells with the NY-ESO-1^−^/HLA-A2^−^ renal cell carcinoma cell line KT-195, which had been transduced either with an HLA-A2 encoding vector or a control vector. While TCR-HL2-tg T cells did not react against KT-195 cells (Fig. 3*b*), TCR-CM26-tg T cells released IFN-γ when they were co-cultivated with HLA-A2-expressing KT-195 cells (668 pg/ml). Compared to the background seen with NY-ESO-1^−^/HLA-A2^+^ melanoma cell lines SK-Mel-29-VC (196 pg/ml) and Me-324 (115 pg/ml) (Fig. 2*d*), the concentration of released IFN-γ was higher for KT-195-A2 cells, probably due to the overexpression of HLA-A2 on these cells. The recognition of KT-195-A2 cells confirmed that TCR-CM26 had an increased recognition of HLA-A2. The CM26 CTL clone was derived from an allogeneic approach, where a T cell population was used which had not been selected against HLA-A2 in the thymus.

## Discussion

We have isolated CTL clones reactive against the cancer-testis antigen NY-ESO-1, which is a promising target antigen for immunotherapies using TCR gene-modified T cells in cancer patients,^7^ and analyzed their TCR for a potential clinical application. For the generation of NY-ESO-1-specific clones, we used different protocols of stimulation: autologous T cells were stimulated with HLA-A2^+^ DC, which were loaded with two different amounts of NY-ESO-1-peptide (NY-ESO-1_157-165_). In a further approach, HLA-A2^−^ T cells were stimulated with allogeneic HLA-A2^+^ DC offering the advantage that the T cell pool had not been selected against HLA-A2/NY-ESO-1 in the thymus.

The CTL clones were compared based on their ability to bind specific MHC-multimers and to react against peptide-loaded T2 cells and tumor cell lines. These experiments resulted in first hints about the quality of the TCR that were expressed by the clones. However, a direct comparison of the TCR was difficult, as the clones might have different cell-intrinsic properties and might also vary in their capability to grow and lyse *in vitro*. For a more meaningful comparison of the different TCR, we isolated their genes and expressed them in parallel as transgenes in primary human T cells derived from the same donor. As evaluation criteria we used preconditions which a TCR has to fulfill in a clinical application: (*i*) efficient expression on TCR-tg T cells, (*ii*) recognition of its antigen with a sufficiently high affinity, so that also endogenously processed antigen can be detected, (*iii*) specificity, so that cells which do not express the targeted antigen are not recognized (no off-target side effects).

The three analyzed TCR were efficiently expressed on TCR-tg T cells when optimized versions (second disulfide bond, minimally murinized and codon-optimized) of the TCR were used. The expression of non-optimized TCR was low for TCR-HL2 and TCR-CM26, indicating that these TCR were weak in terms of cell surface expression.^27^ The specificity and affinity toward the antigenic peptide was first analyzed by multimer binding assays. On all examined cell types, multimer binding was highest for TCR-ThP2, which in addition showed efficient multimer binding on CD8^−^ cells. While the half maximum cytotoxicity of T cell clones ThP2 and HL2 was very similar it differed for TCR-tg T cells by approximately two logs (ThP2: 2 × 10^−7^ M, HL2: 2 × 10^−9^ M). According to this, TCR-ThP2 had the lowest activity in functional assays since only high concentrations of peptide were recognized and TCR-ThP2-tg T cells did not react against any NY-ESO-1^+^ cell line. We hypothesize that there is a threshold affinity level, which determines whether or not a TCR recognizes endogenously presented antigen. As these results indicated that the enhanced multimer binding of TCR-ThP2 was not associated with improved T cell function, it is not sufficient to assess TCR quality based on a single parameter. Similar findings were also reported for other TCR.^28^

The specificity of the TCR was first evaluated by screening a small panel of NY-ESO-1/HLA-A2-expressing and non-expressing tumor cell lines. TCR-HL2- and TCR-CM26-tg T cells secreted high amounts of IFN-γ and showed cytotoxic activity only after co-cultivation with NY-ESO-1^+^/HLA-A2^+^ cells. IFN-γ release and the killing capacity of TCR-CM26-tg T cells was slightly enhanced in comparison to TCR-HL2-tg T cells and we recognized that TCR-CM26-tg T cells secreted small amounts of IFN-γ also in the absence of NY-ESO-1-antigen. To further evaluate the specificity of TCR-HL2 and TCR-CM26, we screened a library consisting of 125 of commonly presented HLA-A2-binding peptides. Although the number of peptides in this library is limited compared to the overall number of HLA-A2-presented peptides on human cells, one can assume that TCR with a broad range of peptide recognition and TCR recognizing an HLA molecule independent of the bound peptide can be identified. Indeed, we found background recognition of NY-ESO-1^−^/HLA-A2^+^ cells by TCR-CM26, which was allusively seen when IFN-γ release was analyzed, but was not observed in cytotoxicity assays. We hypothesize that the affinity of TCR-CM26 to HLA-A2 was too low to result in the killing of target cells when they did not express NY-ESO-1. In further experiments, we got strong indications that the recognition of NY-ESO-1^−^/HLA-A2^+^ cells by TCR-CM26 was due to HLA-A2 alloreactivity. However, we cannot entirely exclude that one or few endogenous peptides presented on T2, KT-195, SK-Mel-29 or Me-324 cells were recognized. But, if endogenous peptides were recognized, the reactivity against unloaded T2 cells should have been stronger compared to peptide-loaded T2 cells, where the endogenous peptides were replaced by the added peptides. For a clinical application it is difficult to predict, whether or not this background recognition of HLA-A2 would cause side effects. Off-target reactivity of TCR, which are generated in an allogeneic priming approach of T cells is not new and was recently described for a large number of WT-1-specific TCR generated in such a setting.^10^ To circumvent this problem, numerous T cell clones must be established for the identification of TCR free of alloreactivity.^8^

In summary, we have molecularly cloned three TCR with specificity for the NY-ESO-1_157-165_ antigenic peptide derived from different approaches of T cell priming. We showed that an extensive evaluation using a combination of different methods is needed to identify a candidate TCR for clinical application. TCR-HL2, which showed an intermediate performance in all functional assays, remains the only candidate for TCR gene therapy. This TCR will now be analyzed in preclinical mouse models.

## Abbreviations

CTL: cytotoxic T lymphocyte
DC: dendritic cell
IFN-γ: interferon-γ
J76: Jurkat76
mAb: monoclonal antibody
MFI: mean fluorescence intensity
NY-multimer: HLA-A2/NY-ESO-1_157-165_-multimer
PBL: peripheral blood lymphocytes
TCR: T cell receptor
TCR-tg: TCR-transgenic
