# Supplementary material for: NY-ESO-1 antigen-reactive T cell receptors exhibit diverse therapeutic capability
Source: Int J Cancer. 2012 Aug 21;132(6):1360–7. doi: 10.1002/ijc.27792 (PMC3617456; doi:10.1002/ijc.27792)
Supplement: Supplementary file 1 [file ijc0132-1360-SD1.doc]

**Suppl. Fig. 1**


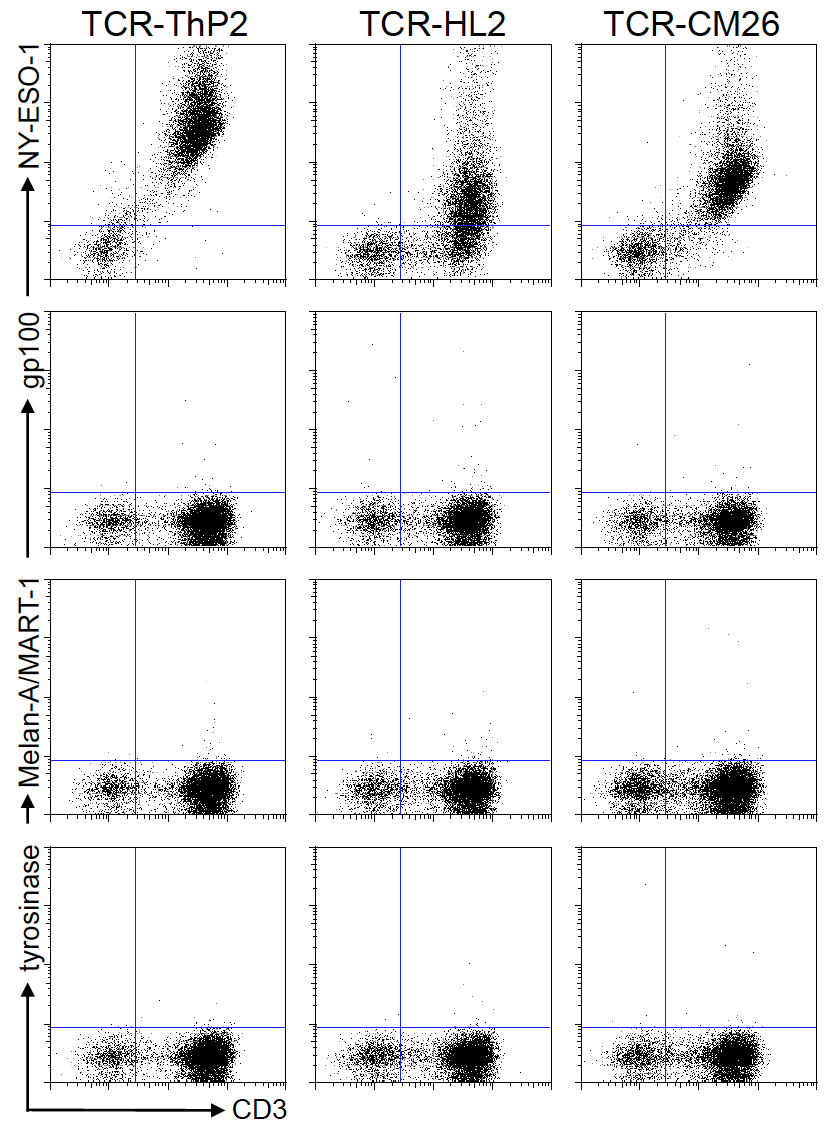


**No unspecific multimer binding by TCR-tg J76/CD8 cells.** J76/CD8 cells were transduced with optimized versions of NY-ESO-1_157-165_-reactive TCR-ThP2, TCR-HL2, and TCR-CM26. Four days after transduction, cells were stained with anti-CD3 mAb and NY-ESO-1-multimers or three different HLA-A2 control multimers (gp100: IMDQVPFSV, Melan-A/MART-1: ELAGIGILTV, and tyrosinase: YMDGTMSQV) and analyzed by flow cytometry.
