# Supplementary material for: NY-ESO-1 antigen-reactive T cell receptors exhibit diverse therapeutic capability
Source: Int J Cancer. 2012 Aug 21;132(6):1360–7. doi: 10.1002/ijc.27792 (PMC3617456; doi:10.1002/ijc.27792)
Supplement: Supplementary file 2 [file ijc0132-1360-SD2.doc]

**Suppl. Fig. 2**

**
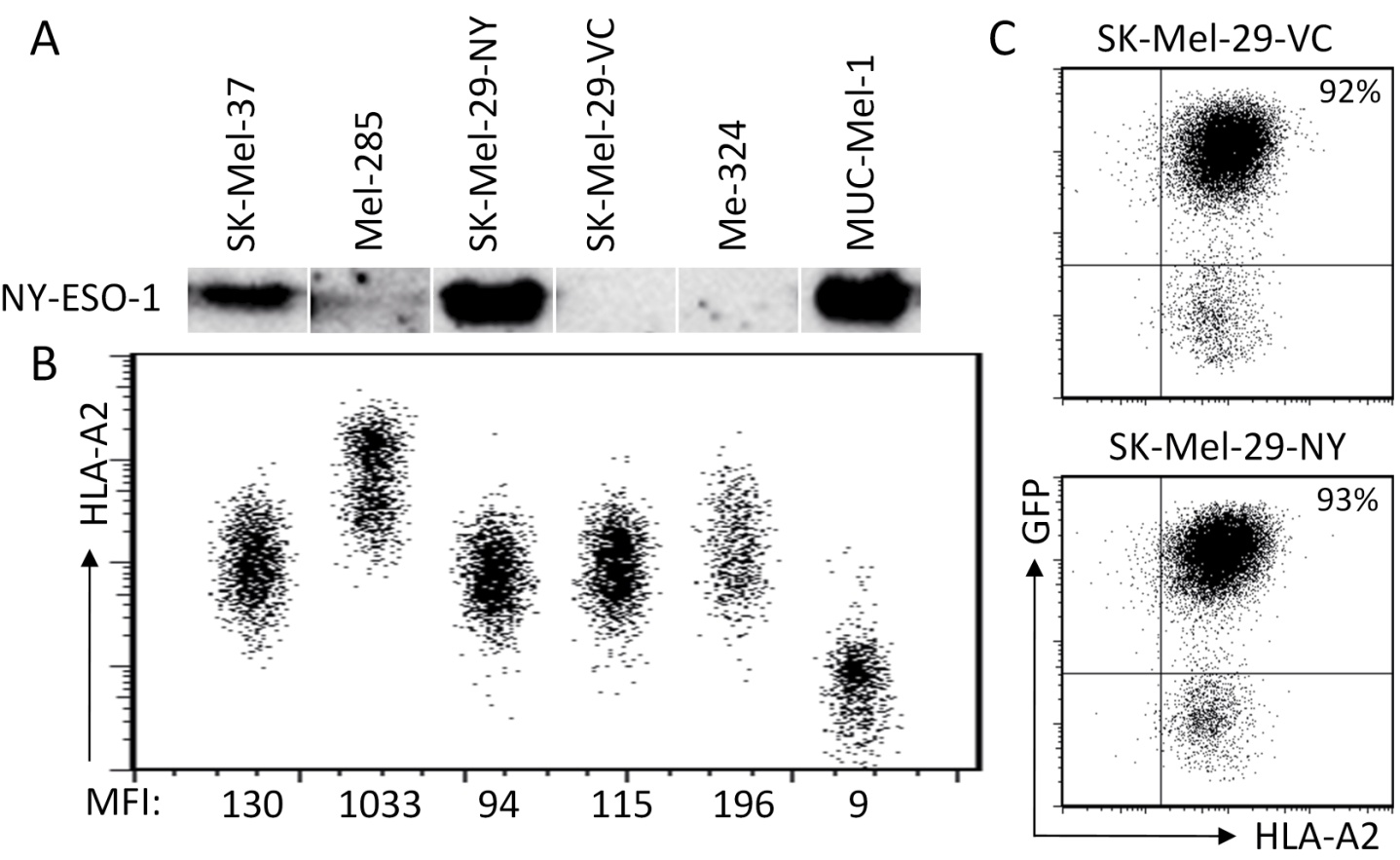
**

**Characterization of target cell lines.** (A) Western blot analysis of NY-ESO-1 protein expression in different melanoma cell lines. (For Mel-285 cells light emission was measured for 5 min instead of 1 min.) (B) Different cell lines were analyzed for HLA-A2 expression by flow cytometry (mAb clone: BB7.2). MFI of HLA-A2 staining are indicated. (C) SK-Mel-29 cells were transduced either with a control vector encoding GFP (VC) or with a vector encoding GFP and NY-ESO-1 (NY). GFP-expressing cells were enriched by FACS. Cells were analyzed for expression of GFP and HLA-A2.
